# Re: Effect of Individual Omega-3 Fatty Acids on the Risk of Prostate Cancer: A Systematic Review and Dose-Response Meta-Analysis of Prospective Cohort Studies

**DOI:** 10.2188/jea.JE20150135

**Published:** 2015-08-05

**Authors:** Shinkan Tokudome

**Affiliations:** Department of Nutritional Epidemiology, National Institute of Health and Nutrition, Tokyo, Japan

Dear Editor-in-Chief,

Recently, Fu et al (JE, 2015;25:261–74)^1^ conducted a meta-analysis based on prospective cohort studies and clarified the association of prostate cancer (PCa) onset with dietary intake and blood concentrations of individual omega-3 fatty acids (or n-3 polyunsaturated fatty acids [PUFAs]). However, they could not find consistent dose-response relationships between PUFA intake/blood levels and PCa risk. Although the authors mentioned possible reasons for this lack of an observed relationship, I would like to add several more.

Their review was based on literature reported from Western countries, largely from the United States, where n-6 PUFA intake is much higher, n-3 PUFA intake much lower, and the ratio of n-6 PUFAs:n-3 PUFAs (or arachidonic acid [AA]:long-chain [LC] n-3 PUFAs) is much higher than in Asian countries/areas (including Japan), as discussed by the researchers.^2^^,^^3^ The results appear to be most generalizable to the people of Western countries where the population shares a common genetic background and lifestyle factors with Western populations.

Furthermore, the authors’ observations may not be biologically plausible. As is well-known, n-6 PUFAs (mainly linoleic acid) are upstream chemicals of the cyclooxygenase pathway, including AA and prostaglandin E2.^4^^,^^5^ On the other hand, n-3 PUFAs (including α-linolenic acid) are precursor substances of LC n-3 PUFAs, such as eicosapentaeoic acid, docosapentaenoic acid, and docosahexaenoic acid. n-6 PUFAs act as inflammatory and carcinogenic chemicals, while n-3 PUFAs are anti-inflammatory and anti-carcinogenic. In addition, not only absolute consumption and blood concentrations of fatty acids but also ratios of n-6 PUFAs:n-3 PUFAs and AA:LC n-3 PUFAs seem crucial for the onset of cancers,^6^^–^^8^ including PCa.^9^

Several epidemiologic aspects of primary prevention should be taken into account. We need to clearly hypothesize etiologic factors in terms of cancer initiators or promoters. Because PCa has a long natural history, it is very difficult to obtain information on etiologic factors from the distant past; this information bias may therefore unduly influence epidemiologic research (case-control studies in particular). Subjects with latent cancer could be misclassified into a control group due to the fact that it is prevalent not only in industrialized countries but also worldwide.

Age and family history are clear risk factors for PCa, but no definite modifiable preventive or risk factors are available to date.^10^ Probable/limited-suggestive (according to the World Cancer Research Fund/American Institute for Cancer Research [WCRF/AICR]) preventive factors are foods containing lycopene, selenium and foods containing selenium, α-tocopherol and foods containing α-tocopherol, and legumes; in contrast, diets high in calcium, milk/dairy products, and processed meat are potentially modifiable risk factors. Relevant antioxidant vitamins and minerals are not specific to PCa but may be applicable for prevention of cancers in general. Legumes are of interest because they contain polyphenols, including isoflavones and daizein (and its metabolite, equol, in particular, which is a biologically active phytoestrogen.)^11^ Milk/dairy products appear critical, not because they are major sources of calcium, but because they contain various growth or promotion factors, including insulin-like growth factor 1 and estrogen. Processed meat appears to be a food representative of an Americanized/Westernized diet and is one of the main sources of not only animal proteins but also iron (a redox radical) and cholesterol (a precursor of steroids, testosterone, and estrogen).^12^

Smoking should not be overlooked, as it is a single potent multi-targeting carcinogen.^13^ Alcohol acts as a carcinogen via alcohol dehydrogenase 1B and acetaldehyde dehydrogenase 2 genetic polymorphisms (GPs).^14^ Physical activity/exercise and participation in sports reduce insulin levels and insulin sensitivity, as well as the risk of diabetes mellitus. Both alcohol consumption and physical activity/sports modify syntheses of steroids, testosterone, and estrogen from cholesterol and change testosterone and estrogen levels and the testosterone:estrogen ratio by way of 5α-reductase GPs.^15^ Intake of vitamin D and sunlight exposure with vitamin D receptor GPs, including Fok-I, may modulate PCa carcinogenesis via cell cycle regulation and apoptosis.

For secondary prevention, controversial findings have been reported among several randomized controlled trials (RCTs) investigating possible PCa mortality-reducing effects of prostate-specific antigen (PSA)-based screening in the European countries^16^ and in the United States.^17^ The conflicting, inconclusive nature of these findings may be due to biases, including selection and information biases, based at least in part on the higher prevalences of PCa and PSA testing in these countries compared to Asian countries. An RCT or case-control study should be conducted to assess the effectiveness and test performance (including sensitivity, specificity, and positive predictive value) of PSA-based screening in Asian countries, which have a similar genetic background and lifestyle factors as well as lower prevalences of PCa and PSA test compared to Western countries.
